# Systematic analysis of alternative splicing signature unveils prognostic predictor for kidney renal clear cell carcinoma

**DOI:** 10.1002/jcp.28840

**Published:** 2019-05-29

**Authors:** Jukun Song, Yong Da Liu, Jiaming Su, Dongbo Yuan, Fa Sun, Jianguo Zhu

**Affiliations:** ^1^ School Of Medicine Guizhou University Guiyang Guizhou China; ^2^ Department of Oral and Maxillofacial Surgery Guizhou Provincial People's Hospital Guiyang Guizhou China; ^3^ Department of Urology, Minimally Invasive Surgery Center The First Affiliated Hospital of Guangzhou Medical University Guangzhou China; ^4^ Guangdong Key Laboratory of Urology Guangzhou China; ^5^ Department of Urology Guizhou Provincial People's Hospital Guiyang Guizhou China

**Keywords:** alternative splicing, kidney renal clear cell carcinoma, The Cancer Genome Atlas

## Abstract

There is growing evidence that alternative splicing (AS) plays an important role in cancer development. However, a comprehensive analysis of AS signatures in kidney renal clear cell carcinoma (KIRC) is lacking and urgently needed. It remains unclear whether AS acts as diagnostic biomarkers in predicting the prognosis of KIRC patients. In the work, gene expression and clinical data of KIRC were obtained from The Cancer Genome Atlas (TCGA), and profiles of AS events were downloaded from the SpliceSeq database. The RNA sequence/AS data and clinical information were integrated, and we conducted the Cox regression analysis to screen survival‐related AS events and messenger RNAs (mRNAs). Correlation between prognostic AS events and gene expression were analyzed using the Pearson correlation coefficient. Protein‐protein interaction analysis was conducted for the prognostic AS‐related genes, and a potential regulatory network was built using Cytoscape (version 3.6.1). Meanwhile, functional enrichment analysis was conducted. A prognostic risk score model is then established based on seven hub genes (KRT222, LENG8, APOB, SLC3A1, SCD5, AQP1, and ADRA1A) that have high performance in the risk classification of KIRC patients. A total 46,415 AS events including 10,601 genes in 537 patients with KIRC were identified. In univariate Cox regression analysis, 13,362 survival associated AS events and 8,694 survival‐specific mRNAs were detected. Common 3,105 genes were screen by overlapping 13,362 survival associated AS events and 8,694 survival‐specific mRNAs. The Pearson correlation analysis suggested that 13 genes were significantly correlated with AS events (Pearson correlation coefficient >0.8 or <−0.8). Then, We conducted multivariate Cox regression analyses to select the potential prognostic AS genes. Seven genes were identified to be significantly related to OS. A prognostic model based on seven genes was constructed. The area under the ROC curve was 0.767. In the current study, a robust prognostic prediction model was constructed for KIRC patients, and the findings revealed that the AS events could act as potential prognostic biomarkers for KIRC.

## 1 INTRODUCTION

In recent years, kidney cancer (KC) has been recognized as a highly malignant tumor of the urinary system, and its incidence rate ranks 11th and 15th among common malignant tumors in men and women, respectively, accounting for 2.2% of all new cancers and 1.8% of all cancer‐related death (Bray et al., 2018; Siegel, Miller, & Jemal, 2018). Kidney cancer includes many different types of kidney tumors (Linehan, 2012). Among them, kidney renal clear cell carcinoma (KIRC) accounts for about 85% of adult kidney malignancies. It is the most common type of kidney malignancy. In 2018, approximately 403,262 new cases and 175,098 deaths were estimated to be associated with KC worldwide (Bray et al., 2018). The efficacy of immunotherapy and molecular targeted drugs is not satisfactory, and the molecular pathologic mechanism of renal cancer remains unclear (Cancer Genome Atlas Research, 2013). Due to resistance to radiotherapy and chemotherapy, surgery is the primary treatment choice for localized renal cancer. Therefore, it is an urgent need to screen potential diagnostic biomarkers or therapeutic targets for the treatment of KIRC.

Protein diversity produces biodiversity in eukaryotic cell biology. The pre‐messenger RNA (mRNA) produced by alternative splicing (AS) is a universal mechanism for generating mRNA isoforms from limited genomes (Lee & Rio, 2015; Nilsen & Graveley, 2010). The human body regulates AS patterns to produce a variety of protein isoforms to meet the needs of complex biological evolution. AS occurs in the majority of human multi‐exon genes, through a process where introns are deleted and it selectively includes or excludes specific exons (Narayanan, Singh, & Shukla, 2017; Salton & Misteli, 2016). Beyond protein diversity, translation of mRNA isoforms can also be disrupted by the introduction of the premature stop codon. Therefore, AS functions as an indispensable role in the biological process, and alterations in splicing patterns often affect the protein function.

There is growing evidence that there is a strong interaction between AS events and tumorigenesis (El Marabti & Younis, 2018; Munkley, Livermore, Rajan, & Elliott, 2017). AS exerts a vital function in the modification of mRNA, especially for specific genes involved in tumor occurrence (Klinck et al., 2008; Kozlovski, Siegfried, Amar‐Schwartz, & Karni, 2017). During the process of different types of AS events, it yielded differences in mRNA levels in various tumors. These proteins play a vital role in cancer‐related biological processes, which are involved in RNA processing, cell proliferation, cell cycle progression, and migration (El Marabti & Younis, 2018).

In the work, a combination of bioinformatic analyses was used to screen the prognostic AS events/genes in KIRC. The Cox regression analyses were used to select the prognostic AS events and genes. A regulatory network of AS events in KIRC was established and the potential mechanisms were explored. Seven hub genes (KRT222, LENG8, APOB, SLC3A1, SCD5, AQP1, and ADRA1A) were identified which acted as diagnostic biomarkers for forecasting the prognosis of KIRC. Our results provide the basis for further investigations into the pathomechanisms of KIRC and selection of the potential biomarkers for the early diagnosis of KIRC.

## 2 MATERIALS AND METHODS

### 2.1 Data collection of AS events and data processing

We downloaded the RNA sequencing profiles (level 3) from The Cancer Genome Atlas (TCGA) data portal of the KIRC cohort (https://tcga‐data.nci.nih.gov/tcga/). In addition, we collected data of AS events from the TCGA SpliceSep (https://bioinformatics.mdanderson.org/TCGASplice‐Seq/). The percent splicing (PSI) values ranging from 0 to 1 are typically used to quantify AS events (Ryan, Cleland, Kim, Wong, & Weinstein, 2012), and we calculated PSI values for each type of AS events. The 72 nontumor samples and 533 KIRC samples were enrolled in the analysis of AS events, while 72 nontumor samples and 529 KIRC samples were included in the mRNA data set. We also downloaded fully clinical follow‐up information data of 537 patients. from TCGA KIRC cohort.

**Figure 1:**
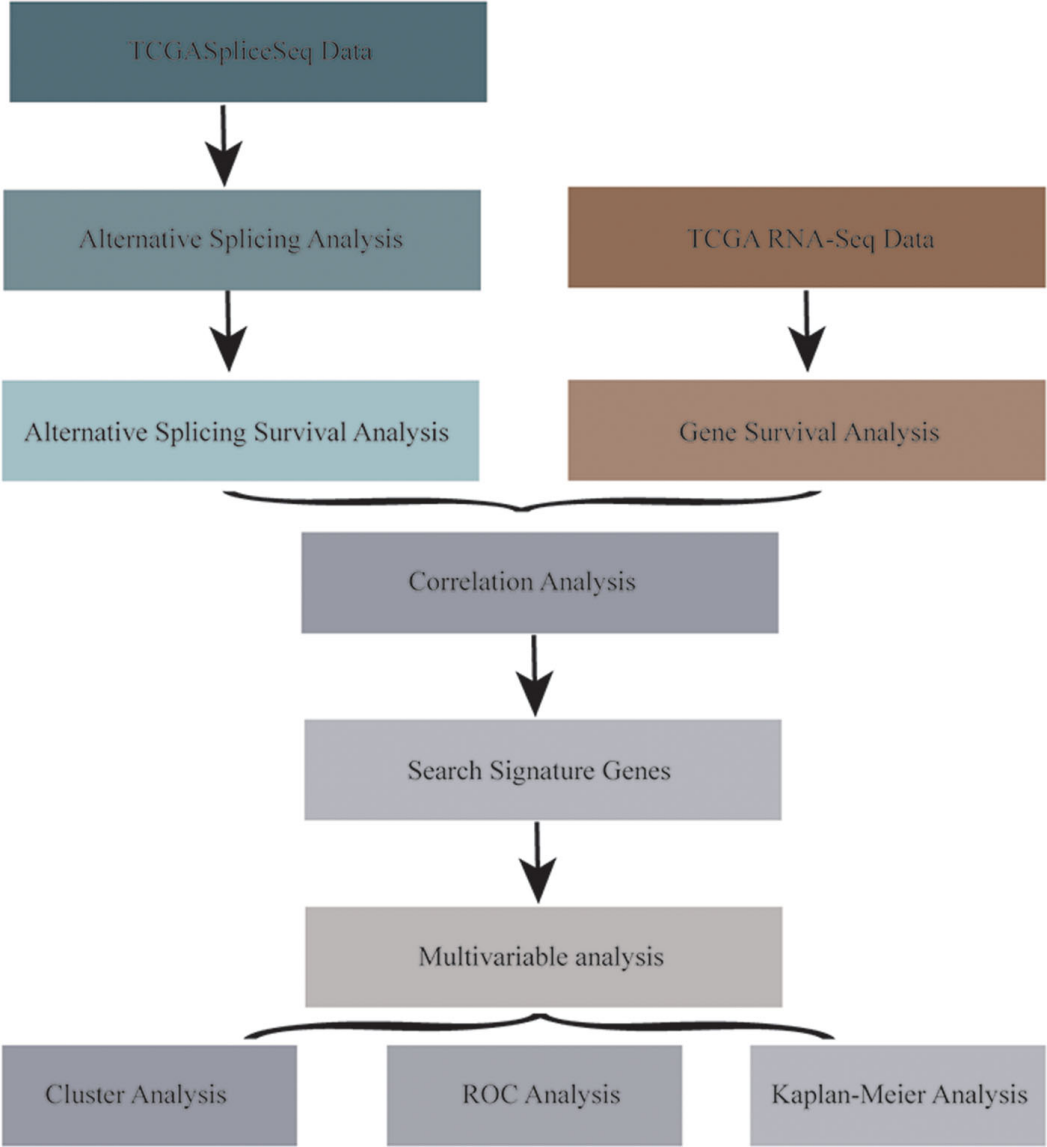
The flowchart of model construction. ROC, receiver operating characteristic; TCGA, The Cancer Genome Atlas [Color figure can be viewed at wileyonlinelibrary.com]

The RNA‐Seq expression datasets were downloaded and then transformed from Fragments PerKilobase Million (FPKM) data into Transcripts PerKilobase Million (TPM) data. Using GRCh38.p2 annotation information provided by GENCODE, Ensemble ID was transformed into gene symbol, and the encoded protein gene was obtained. Finally, 605 samples with AS and RNA‐Seq data were included in the study, and 19,814 genes were obtained. The flowchart of the model building is shown in Figure 1.

### 2.2 Distribution of AS events

**Figure 2:**
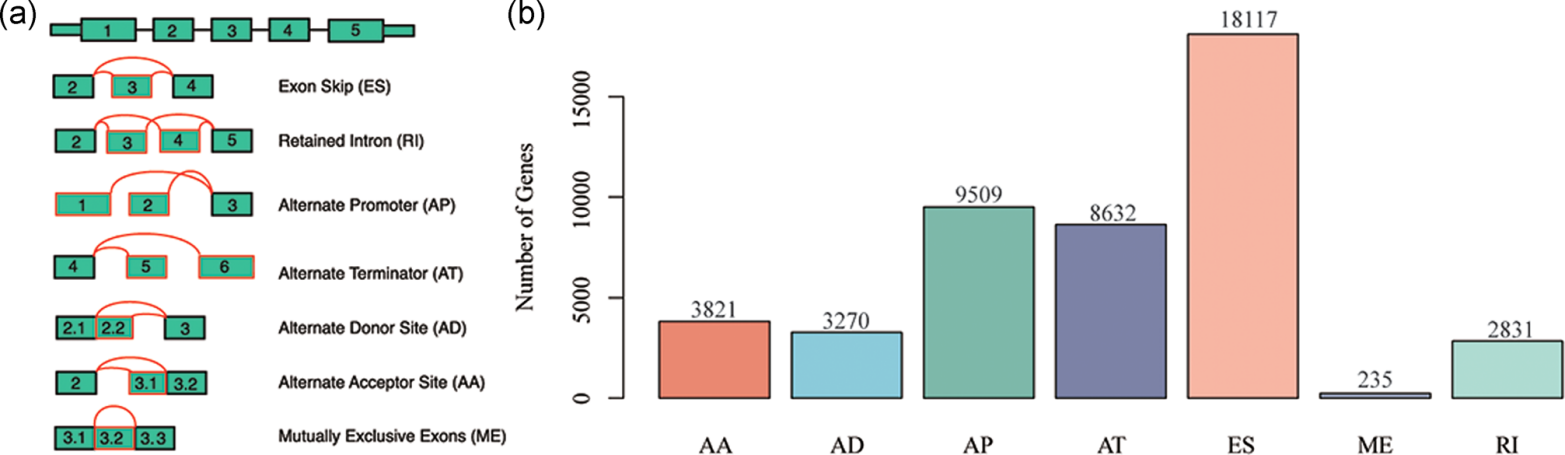
Characterization of seven types of AS in the study. (a) Typical patterns for seven types of AS events; (b) Number of AS events/mRNAs from the 605 KIRC patients. AA, alternate acceptor site; AD, alternate donor site; AP, alternate promoter; AS, alternative splicing; AT, alternate terminator; ES, exon skip; ME, mutually exclusive exons; RI, retained intron; KIRC, kidney renal clear cell carcinoma [Color figure can be viewed at wileyonlinelibrary.com]

There are seven types of AS events, including retained intron (RI), exon skip (ES), alternate promoter (AP), mutually exclusive exons (ME), alternate donor site (AD), alternate terminator (AT), and alternate acceptor site (AA). We analyzed the distribution of protein‐coding genes in seven types of AS events in KIRC. A visualization plot for AS events is shown in Figure 2.

### 2.3 Screening for prognostic AS events and mRNAs

**Figure 3:**
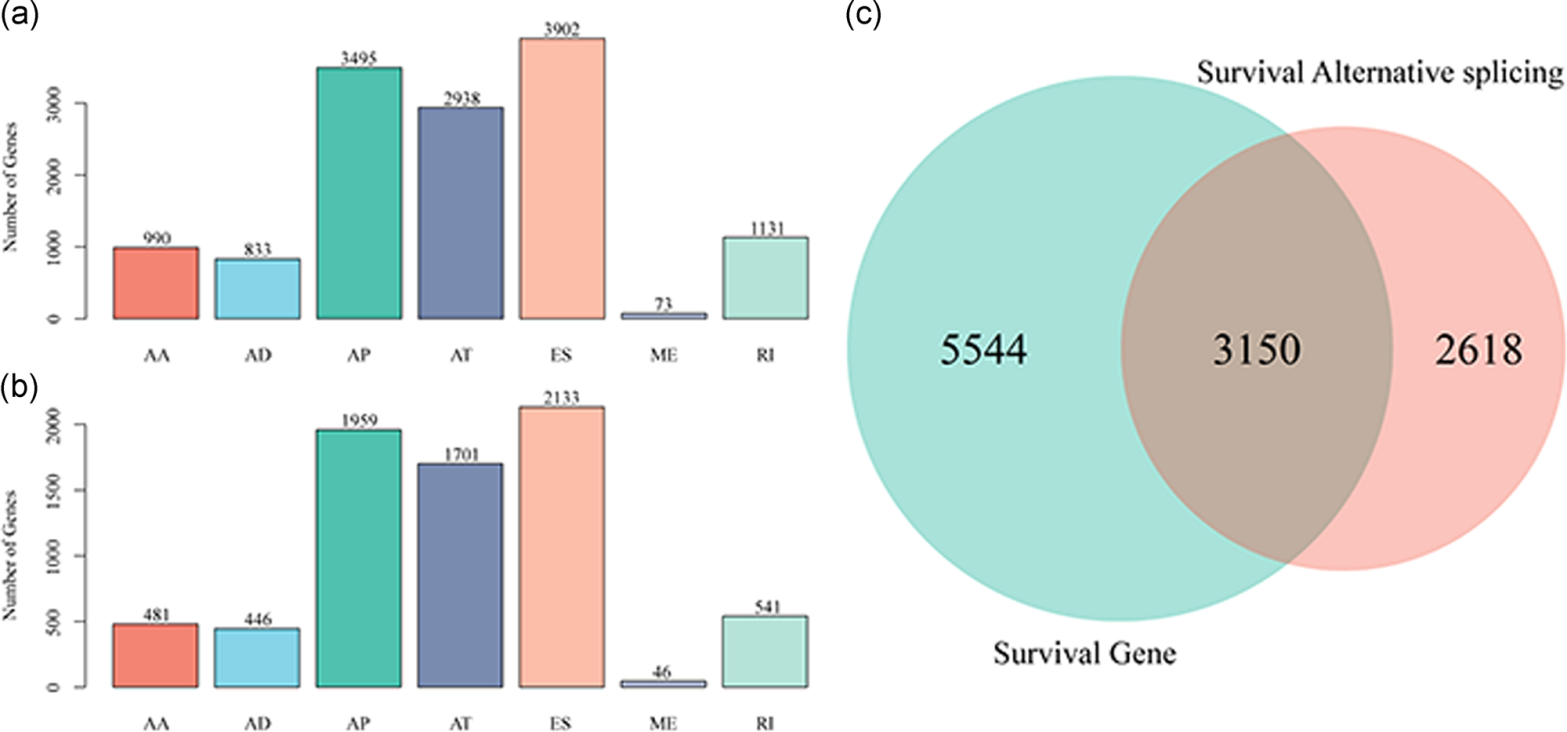
Number of prognostic AS events/mRNAs. (a) Histograms of the seven types of prognostic AS events. (b) Histogram of prognostic mRNAs among seven types of AS events. (c) The Venn diagram exhibits the intersection of prognostic AS events/mRNAs. AA, alternate acceptor site; AD, alternate donor site; AP, alternate promoter; AS, alternative splicing; AT, alternate terminator; ES, exon skip; mRNA, messenger RNA; ME, mutually exclusive exons; RI, retained intron [Color figure can be viewed at wileyonlinelibrary.com]

Survival‐related AS events/mRNAs with *p* < .05 were determined using univariate Cox regression analysis. The distribution of the survival‐specific AS events/mRNAs was visualized in Figure 3. Then, we conducted the multivariate Cox regression analysis to determine the AS events which could serve as independent prognostic biomarkers, and the prediction model was built. To understand whether this model is robust or not, the prognostic performance was assessed by the time‐dependent receiver operating characteristic curves (Heagerty, Lumley, & Pepe, 2000).

### 2.4 UpSet plot and construction of gene interaction network

When dealing with five or more groups, the Upset diagram was generated by the UpSetR package (version 1.3.3) (Lex, Gehlenborg, Strobelt, Vuillemot, & Pfister, 2014) instead of the traditional Venn diagram to determine the association between the interaction sets. To visualize the regulatory network interactions among prognostic genes in AS events, we mapped these genes into a String database to obtain interactions using scores >0.4 and visualized them using Cytoscape (version 3.6.1).

### 2.5 Gene Ontology and KEGG terms enrichment analysis

To better explore the biological processes and pathways associated with the AS‐related genes, the functional and pathway enrichment analysis was used with the R package “ClusterProfiler” (Yu, Wang, Han, & He, 2012). The Kyoto Encyclopedia of Genes and Genomes (KEGG) functional pathway analysis was visualized using Cytoscape (version 3.6.1). A *p* < .05 was considered statistically significant.

### 2.6 Establishment of the prognostic model

We conducted the multivariate Cox regression analyses to assess whether the prognostic genes in the seven types of AS events could serve as independent prognostic biomarkers for overall survival (OS). We used a combination of gene expression levels weighted by regression coefficient (β) originating from the multivariate Cox regression analysis to construct a risk score model. The formula for estimating the risk score for each patient is as follows: Risk score = β_gene1_ × expr_gene1_ + β_gene2_ × expr_gene2_ + ··· + β_genen _×_ _expr_genen_. A *p* < .05 was considered statistically significant.

## 3 RESULTS

### 3.1 Overview of AS events in KIRC

The profile of AS events/genes for 487 patients from TCGA KIRC cohort was analyzed. After analyzing raw data, 46,415 AS events were detected, including 235 ME in 229 genes, and 2,831 RI in 1,904 genes, 3,720 AD in 2,302 genes, 3,872 AA in 2,685 genes, 8,632 AT in 3,772 genes, 9,509 AP in 3,807 genes, and 18,117 ES in 6,917 genes (Figure 2). Among the seven types of AS events, ES is the main AS type, and the rarest AS type is ME. The results indicate that there may be several AS models for a gene.

### 3.2 Prognostic AS events in the KIRC cohort

To explore the prognostic strength of the different type of AS events, we performed a univariate Cox regression analysis. The results showed that 13,362 AS events, including 5,768 genes, significantly correlated with OS with a *p* < .05 (Figure 3a). In addition, we performed univariate Cox regression analysis to identify survival‐specific mRNAs. As a result, we found that 8,694 out of 19,754 mRNAs in seven types of AS events were considered as survival‐related genes with the threshold of *p* < .05 (Figure 3b). As shown in Figure 3, the number of prognostic ES events obviously reduced, whereas AP events increased. These findings demonstrated that the majority of ES events were not related to survival, but some AP events correlated with prognosis. The common 3,105 genes were further filtered by intersecting 5,768 AS associated genes and 8,694 mRNAs (Figure 3c).

**Figure 4:**
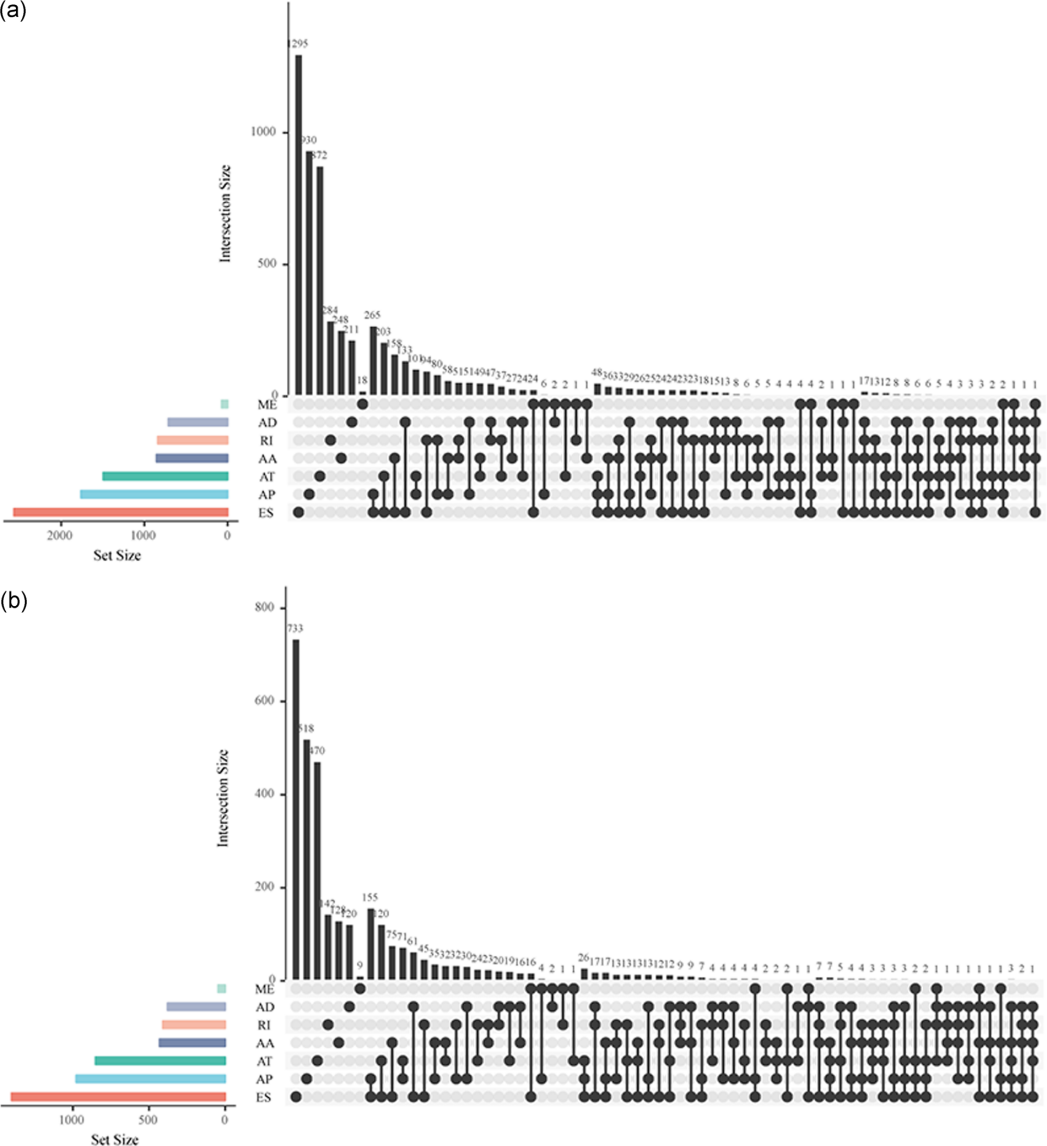
UpSet plot shows the distribution of seven types of prognostic AS events/genes. (a) The distributions of seven different types of AS‐related genes that significantly correlated with overall survival. (b) The distributions of seven different types of AS‐related genes that significantly correlated with gene expression. AA, alternate acceptor site; AD, alternate donor site; AP, alternate promoter; AS, alternative splicing; AT, alternate terminator; ES, exon skip; ME, mutually exclusive exons; RI, retained intron [Color figure can be viewed at wileyonlinelibrary.com]

AS leads to the expression of multiple RNA and protein isoforms from one gene, and hence it is responsible for protein diversification in eukaryotes. The prognosis associated AS events were selected. The distribution of genes in such events is shown in Figure 4. From the figure, it can be inferred that a specific gene may possess multiple types of AS events. The types of AS events and mRNAs may be related to the prognosis.

**Figure 5:**
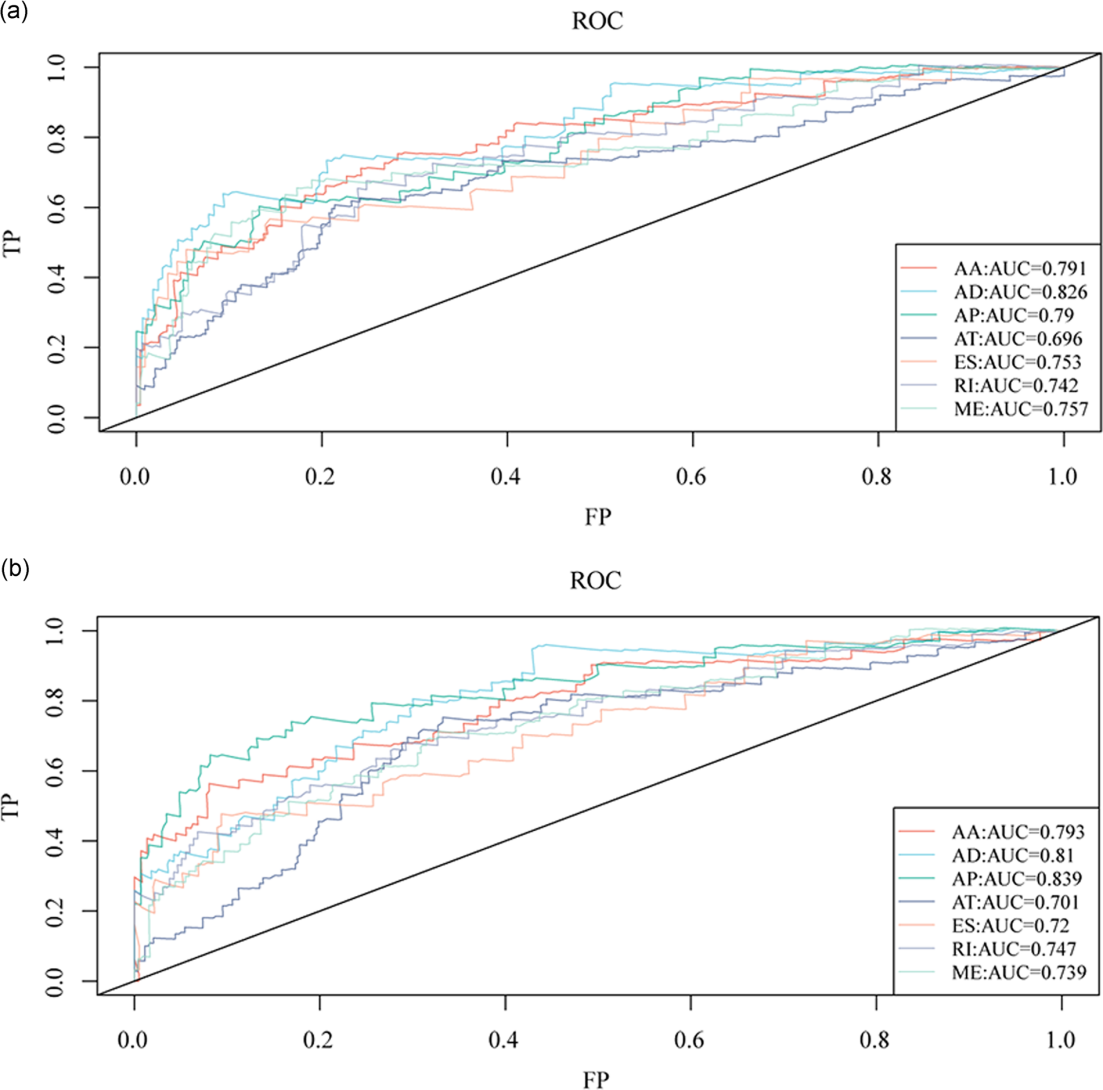
ROC curves of risk score models constructed for KIRC patients. (a) The AUC curve of prognostic classification for the top 30 genes among seven different types of prognostic AS events. (b) The AUC curves for prognostic classification for the top 30 genes in 3,150 prognostic genes were related to AS events. AA, alternate acceptor site; AD, alternate donor site; AP, alternate promoter; AS, alternative splicing; AUC, area under the curve; AT, alternate terminator; ES, exon skip; ME, mutually exclusive exons; RI, retained intron; KIRC, kidney renal clear cell carcinoma; ROC, receiver operating characteristic [Color figure can be viewed at wileyonlinelibrary.com]

To assess the prognostic effects of different AS events on KIRC prognosis, we selected the top 30 genes in each group of seven types of AS events as subjects and performed multivariate Cox regression analysis to identify prognostic biomarkers and establish prediction model. In KIRC, AA, AD, AP, AI, ES, RI, and ME had an area under the curve (AUC) >0.6, and AD had the highest performance in the risk classification of KIRC patients (AUC: 0.810–0.826) (Figure 5).

### 3.3 Functional enrichment analysis

**Figure 6:**
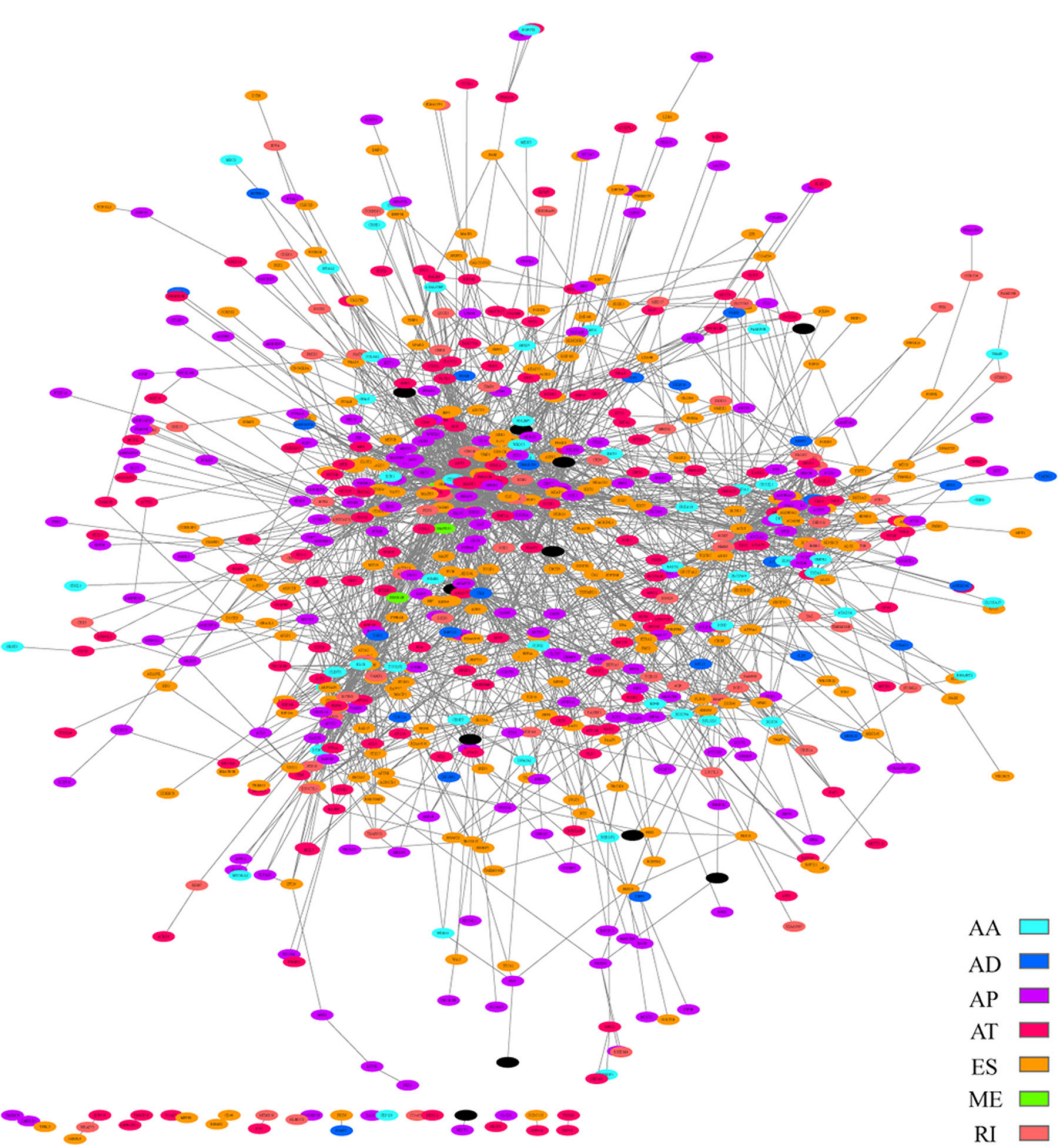
Gene interaction networks of survival associated seven types of AS events generated by Cytoscape. AA, alternate acceptor site; AD, alternate donor site; AP, alternate promoter; AS, alternative splicing; AT, alternate terminator; ES, exon skip; ME, mutually exclusive exons; RI, retained intron [Color figure can be viewed at wileyonlinelibrary.com]

**Figure 7:**
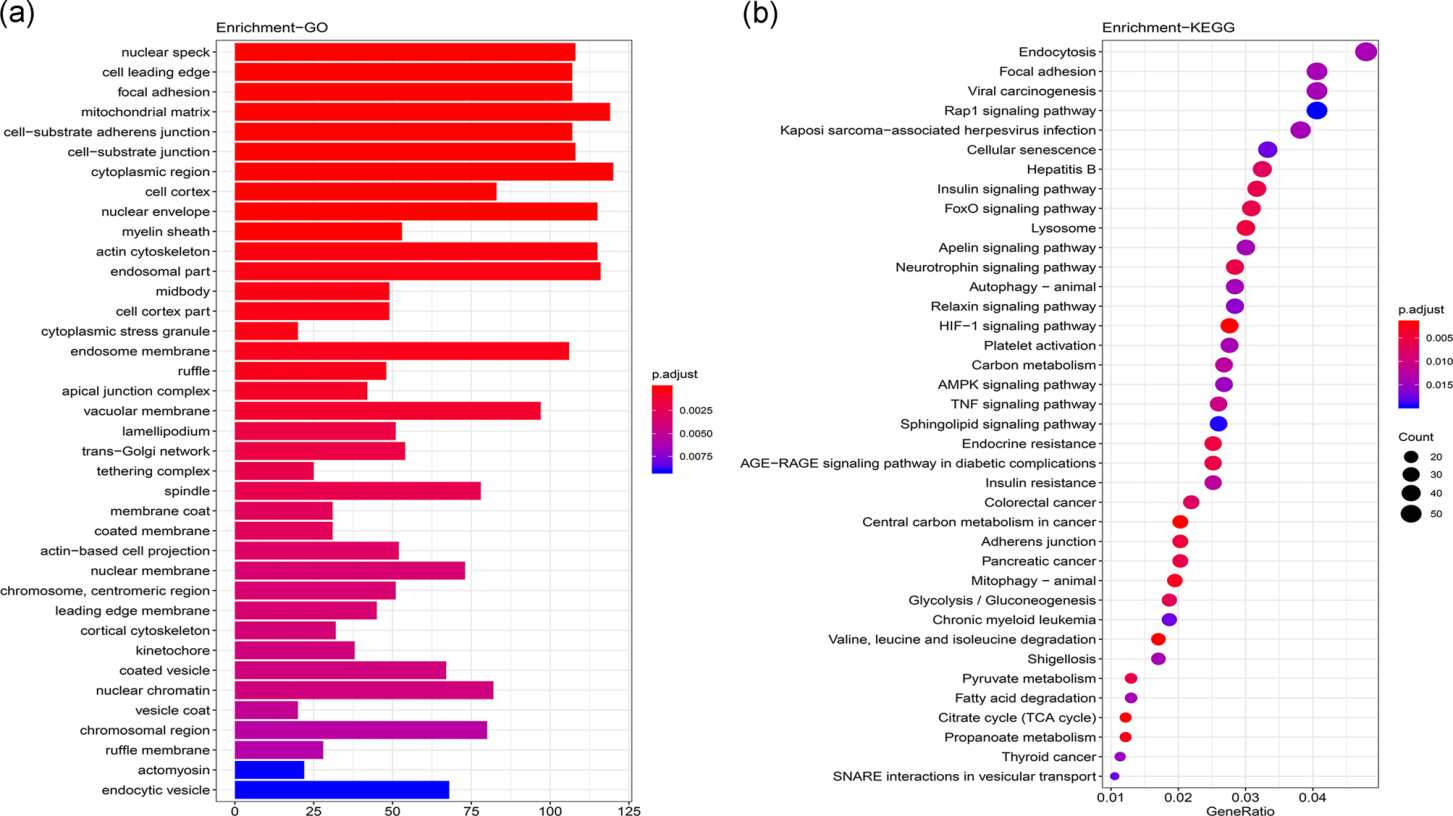
The top 38 categories of enrichment analysis. (a) GO (b) KEGG. GO, Gene Ontology; KEGG, Kyoto Encyclopedia of Genes and Genomes [Color figure can be viewed at wileyonlinelibrary.com]

A protein‐protein interaction network was constructed, and gene interaction networks of prognostic seven types of AS events were generated by Cytoscape (Figure 6). The Gene Ontology (GO) categories and KEGG pathways for survival‐related AS genes were performed. In the GO analysis, 1,227 GO categories were detected. The AS genes were enriched in cellular functions, such as cell leading edge, nuclear speck, cell‐matrix adhesion junctions, and cell cortex (Figure 7a). In the KEGG analysis, 57 KEGG pathways were enriched in HIF‐1 signaling pathway (hsa04066), AGE‐RAGE signaling pathway in diabetic complications (hsa04933), TNF signaling pathway (hsa04668), and Autophagy (hsa04140) (Figure 7b).

### 3.4 Network construction for prognostic AS events

**Figure 8:**
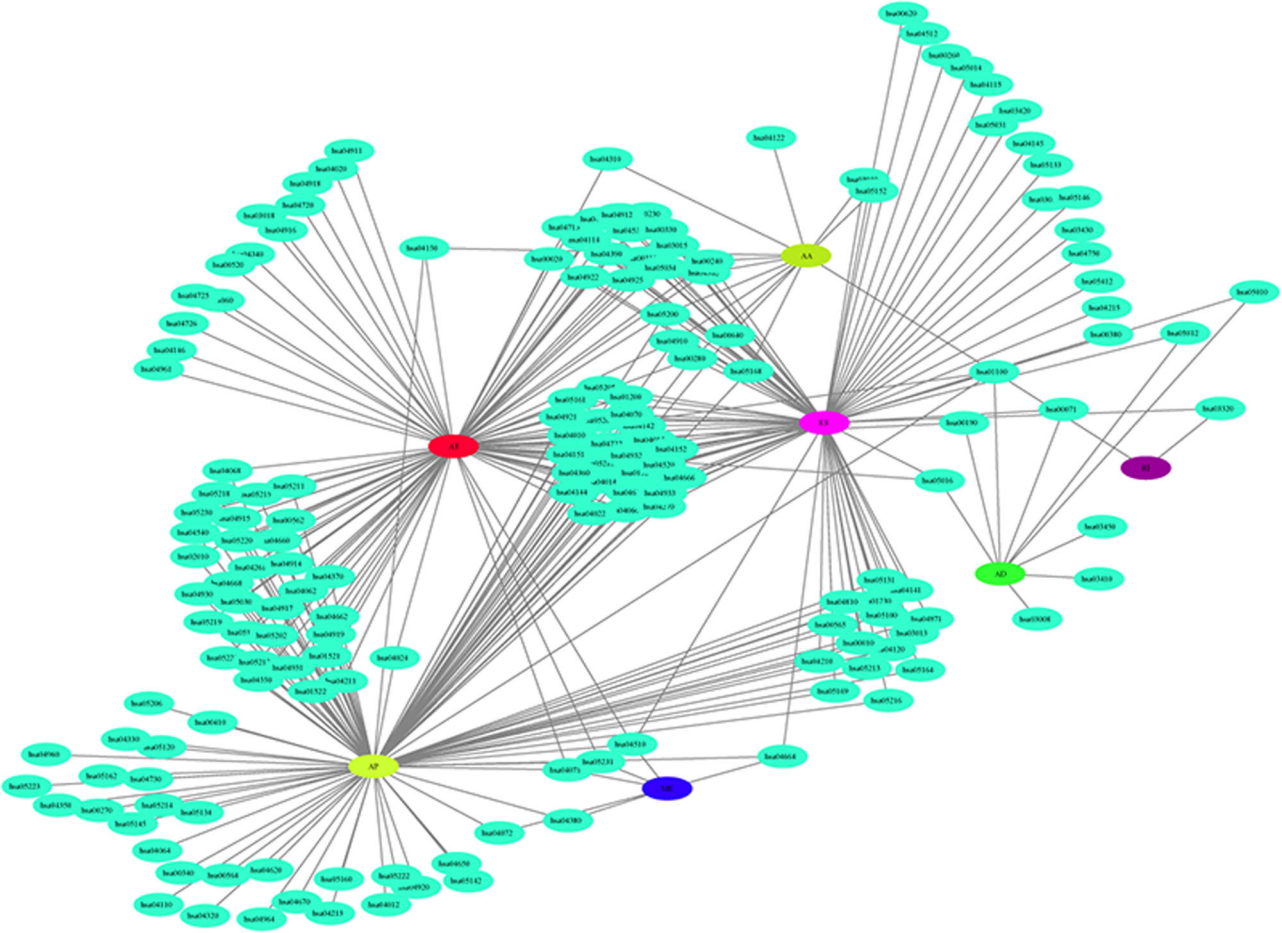
Regulatory network of KEGG pathways for prognostic mRNAs among seven types of AS events. AS, alternative splicing, KEGG, Kyoto Encyclopedia of Genes and Genomes; mRNA, messenger RNA [Color figure can be viewed at wileyonlinelibrary.com]

To explore the interactions among prognostic genes in seven types of AS events, the most significant prognostic genes were selected (*p* < .01) and mapped to a String database with a score >0.4. The interactions among these genes were obtained. Visualization using Cytoscape is presented in Figure 8. The findings show that the majority of prognostic genes in the AS events displayed protein‐protein interactions. This suggests that most of these genes participate in different biological processes.

### 3.5 Establishment of a prognostic prediction model for KIRC

**Figure 9:**
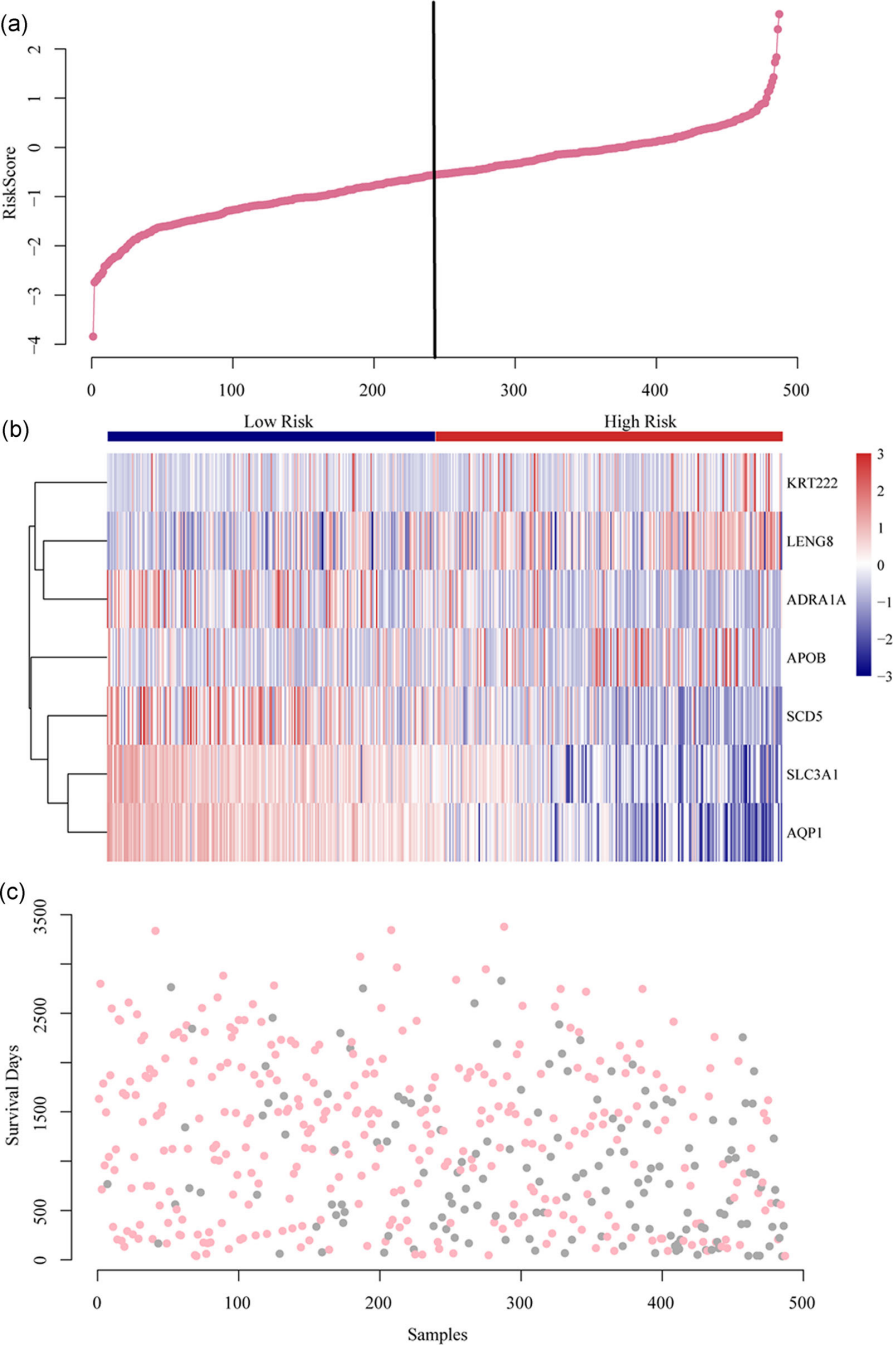
Establishment of prognostic risk score models. (a) Risk score plot. (b) Heatmap of prognostic genes among seven types of AS events. The figure shows that the expression of each gene is significantly different between high and low‐risk groups. (c) Survival time and status for each KIRC patient. AS, alternative splicing; KIRC, kidney renal clear cell carcinoma [Color figure can be viewed at wileyonlinelibrary.com]

The OS range for patients with KIRC was 36 to 3,377 days, and the median OS was 1,102 days. The 13 hub genes with a Pearson correlation coefficient >0.8 or <−0.8 were selected, and these genes correlated with the survival of KIRC patient. Finally, seven hub genes (KRT222, LENG8, APOB, SLC3A1, SCD5, AQP1, and ADRA1A) were identified from the Cox multivariate analysis (Table 1). The seven genes were significantly associated with AS events and were chosen as hub genes to determine the prognostic biomarkers for KIRC patients (Table 1). With the seven hub genes, the risk score model was constructed (Figure 9).

**Table 1:** Seven genes significantly correlated with overall survival in multivariate Cox regression analysis

Gene symbol	Coef	Exp (coef)	Se (coef)	*Z*	*p* value
KRT222	0.349609	1.418513	0.117539	2.97	.0029
LENG8	0.005281	1.005295	0.001274	4.15	3.40E‐05
APOB	0.006542	1.006564	0.003406	1.92	.0547
SLC3A1	−0.0021	0.997902	0.000817	−2.57	.0101
SCD5	−0.003789	0.996218	0.002496	−1.52	.129
AQP1	−0.000622	0.999378	0.000258	−2.41	.016

**Figure 10:**
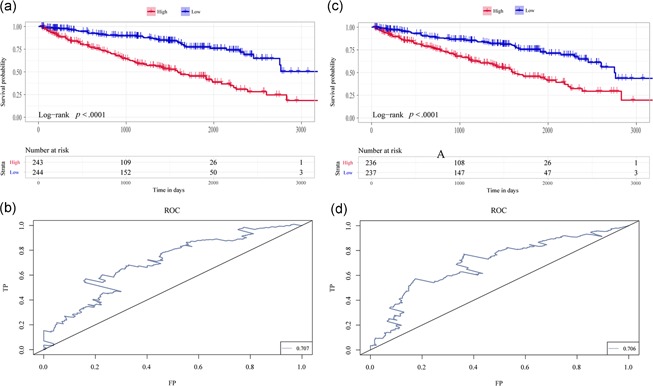
ROC and KM curves of the risk score model based on the seven characteristic genes. Plot (a) and (c) are the KM curves for OS associated AS events and mRNAs, respectively. The curves show that the model can classify the prognosis of KIRC; Plot (b) and (d) are the AUC for OS associated AS events and mRNAs, respectively. It can be seen that the AUC is significantly high. AS, alternative splicing; AUC, area under the curve; KM, Kaplan‐Meier; mRNA, messenger RNA; OS, overall survival; ROC, receiver operating characteristic [Color figure can be viewed at wileyonlinelibrary.com]

To construct and validate the prognostic prediction model for KIRC patients, the seven characteristic genes were used to construct a multifactor survival model and to classify the prognosis in combination with the expression matrix. The results demonstrated that the seven genes had a good prognostic classification power in both datasets, and the AUC was high, suggesting that these genes may serve as prognostic markers for KC (Figure 10).

## 4 DISCUSSION

AS plays an indispensable role in the modiﬁcation of mRNA isoforms, and it permits cells to produce a variety of mRNA and protein isoforms with multiple functions. AS is also responsible for producing biodiversity (El Marabti & Younis, 2018). Abnormal AS is one of the molecular markers of cancer (Oltean & Bates, 2014). Although many cancer‐specific mRNA isoforms have been identified, a systematic overview of AS events and their functional properties has not been conducted. Since the rapid growth of high‐throughput sequencing technology and bioinformatics methods, a comprehensive understanding of AS events in KC is required. In the current study, AS profiles were analyzed and used to construct a regulatory network for KIRC using TCGA data. Several prognostic AS events/genes were identified, which could provide potential treatment targets for KIRC patients.

Recent studies have demonstrated the role of abnormal AS in KIRC (Lehmann et al., 2015; Li et al., 2017a). However, there have been no reports of a comprehensive assessment of the prognostic power of AS events in KIRC. Evidence confirms that AS have vital roles in the occurrence of KIRC. In this study, 10,601 mRNAs in 46,145 AS events in KIRC were observed, suggesting that AS is a common event in the development of KIRC. From the survival analysis, 13,362 survival‐specific AS events for 5768 genes and 8,694 survival associated genes were detected. The common prognostic genes between AS event and genes included KRT222, LENG8, APOB, SLC3A1, SCD5, AQP1, and ADRA1A, which played roles in tumor biology. Based on the seven hub genes, a prognostic model was established. The AUC value exceeded 0.767 when predicting 3,000‐day survival in KIRC patients. The prognostic biomarkers based on AS events can be used to forecast the prognosis of KIRC patients.

Among the seven prognostic genes, KT222 displayed consistent AS changes in various cancers (Li et al., 2017b). Differential expression of LENG8 in breast cancer has been confirmed (Ye et al., 2015). Glycosylated apolipoprotein B (apoB) is a risk factor for the development of myocardial infarction whereas glycosylated apoB is associated with dysplasia and tumor tissue (Reddavide et al., 2011). SLC3A1 was reported to be involved in the occurrence of breast cancer (Jiang et al., 2017). A growing body of evidence suggests that the SCD family plays a key role in coordinating lipid synthesis, energy sensing, and interweaving pathways that influence mitogenesis and tumorigenic transduction signals (Igal, 2010). AQPs play important roles in tumorigenesis, AQP1.4 is associated with lung cancer metastasis (Xie et al., 2012). It has been reported that ADRA1A is highly expressed in peripheral blood vessels of patients with uterine cancer, and ADRA1A regulates proliferation, inhibits tumor formation/metastasis (Al‐Temaimi et al., 2016). These results demonstrate that most genes play a crucial role in the development of cancer.

By analyzing the relationship between AS events/genes and patient's prognosis, we found that AS events may be predictors of KIRC prognosis. Seven potential genes (KRT222, LENG8, APOB, SLC3A1, SCD5, AQP1, and ADRA1A) were identified from the interaction network and correlation analysis between AS event and gene expression. Many of these genes were involved in the development of cancer. From the multivariate analysis for survival, the seven genes may be used to become biomarkers in classification for KIRC prognosis at AS events and gene expression levels.

## CONFLICT OF INTERESTS

The authors declare that there is no conflict of interests.

## AUTHOR CONTRIBUTIONS

JKS, DBY, JGZ, and FS wrote the main manuscript text. JKS and YDL prepared Figures 1 to 10. JMS and JGZ contributed to data analysis. All authors reviewed the manuscript.
